# Peripheral blood mononuclear cell mitochondrial copy number and adenosine triphosphate inhibition test in NAFLD

**DOI:** 10.3389/fendo.2022.967848

**Published:** 2022-10-24

**Authors:** A-Hyeon Lee, Ju Hee Oh, Hyun Sung Kim, Jeong-Hun Shin, Eileen L. Yoon, Dae Won Jun

**Affiliations:** ^1^ Department of Translational Medicine, Hanyang University Graduate School of Biomedical Science and Engineering, Seoul, South Korea; ^2^ Department of Pathology, Hanyang University School of Medicine, Seoul, South Korea; ^3^ Internal Medicine, Hanyang University School of Medicine, Seoul, South Korea

**Keywords:** NAFLD, mitochondrial dysfunction, mitochondria copy number, ATP, biomarker

## Abstract

**Background and aim:**

Non-alcoholic fatty liver disease (NAFLD) is associated with mitochondrial dysfunction. This study aims to develop biomarkers for assessing mitochondrial dysfunction in patients with NAFLD.

**Methods:**

Mitochondrion-associated transcriptome analysis was performed. Peripheral blood mononuclear cells obtained from patients with NAFLD (69) and healthy controls (19) were used to determine the mitochondrial DNA (mtDNA) copy number. A mitochondrial inhibition substrate test (ATP assay) was performed in HepG2 cells using the patient serum.

**Results:**

Hepatic mRNA transcriptome analysis showed that the gene expression related to mitochondrial functions (mitochondrial fusion, apoptotic signal, and mitochondrial envelope) increased in patients with steatohepatitis, but not in those with NAFL. Gene set enrichment analysis revealed that the upregulated expression of genes is related to the pathways of the tricarboxylic (TCA) cycle and deoxyribonucleic acid (DNA) replication in patients with steatohepatitis, but not in healthy controls. The mtDNA copy number in the peripheral blood mononuclear cells was 1.28-fold lower in patients with NAFLD than that in healthy controls (*P* <.0001). The mitochondrial inhibition substrate test showed that the cellular adenosine triphosphate (ATP) concentration was 1.2-fold times less in NAFLD patients than that in healthy controls (*P* <.0001). The mtDNA copy number and mitochondrial ATP inhibition substrate test demonstrated negative correlations with the degree of hepatic steatosis, whereas the ATP concentration showed a positive correlation with the mtDNA copy number.

**Conclusion:**

The mitochondrial copy number of peripheral blood mononuclear cells and mitochondrial ATP inhibition substrate can be used as biomarkers for assessing the mitochondrial dysfunction in patients with NAFLD.

## Introduction

Approximately 25% of the global population is afflicted with non-alcoholic fatty liver disease (NAFLD) (1). NAFLD, characterized with excessive accumulation of lipids in the liver, is the most common cause of chronic liver diseases (2). The mitochondrion plays a critical role in the oxidation of lipids in the liver and also serves as a source of stimuli for oxidative stress, which is closely related to the oxidation of accumulated fats (3). NAFLD is closely related with mitochondrial dysfunction, which is further correlated with diabetes. Apart from inducing fat accumulation, mitochondrial dysfunction may also mediate the production of cytokines that contribute to the progression of NAFLD through fibrosis, hepatitis, and generation of reactive oxygen species (ROS) (4). ROS may be involved in the destruction of the electron-transport chain and/or the transparency of the outer mitochondrial membrane in NAFLD.

Many previous studies on patients with NAFLD have reported varying levels of ultrastructural mitochondrial abnormality, reduced activity of the respiratory chain, ATP depletion, increased outer and inner mitochondrial membrane transparency, and a fall in the mitochondrial DNA (mtDNA) copy number mediated by the overproduction of ROS and oxidative stress (5, 6). In NAFLD, oxidative stress causes mitochondrial dysfunction, which in turn reduces fatty acid levels in the liver and enhances the level of hepatic steatosis and contributes to a vicious cycle that further increases the mitochondrion-induced oxidative stress (7). However, relevant clinical biomarkers are yet to be established to evaluate mitochondrial dysfunction in the NAFLD. Although a few previous studies have reported a method that utilizes the drop in the intracellular ATP concentration to assess mitochondrial dysfunction caused by endocrine-disrupting chemicals (EDCs) (8), a mitochondrial ATP inhibition substrate test for application in the NAFLD has not yet been investigated.

The mtDNA copy number is a biomarker of mitochondrial function. The levels of the mtDNA copy number have been reported to be associated with the overall mortality and several age-related diseases, including cardiovascular diseases, chronic kidney disease, and various cancers (9). A previous study has revealed that patients with NAFLD have fewer mtDNA copy numbers in their peripheral blood mononuclear cells compared to those in control subjects (10).

However, it is still unclear whether the peripheral mtDNA copy number and the mitochondrial ATP inhibition substrate can be used as a non-invasive biomarker to evaluate mitochondrial dysfunction in NAFLD. The present study aims to develop a non-invasive biomarker to investigate mitochondrial dysfunction in patients with NAFLD.

## Methods

### Human study design

This prospective case–control study was conducted with the approval of the Institutional Review Board at Hanyang University Medical Center (IRB: 2020-06-048-004, 2019-12-028-006). A signed voluntary consent was obtained from each patient.

### Inclusion and exclusion criteria

Patients who visited the health examination center and gastroenterology department of Hanyang University Medical Center for health checkup and chronic liver disease were enrolled as the study subjects. Among the participants, 43 patients had undergone liver biopsy. NAFLD patients diagnosed with hepatic steatosis through ultrasound but without any history of significant alcohol consumption (<210 g/week for male patients and <140 g/week for female patients) and any other chronic liver inflammation or hepatitis type B or C were included. Pregnant and/or lactating mothers were excluded.

### Assessment of hepatic steatosis and fibrosis

The transient elastography (TE, FibroScan^®^) test was used to assess hepatic steatosis and fibrosis. Hepatic steatosis was measured through CAP (controlled attenuation parameter) expressed in dB/m. Fibrosis was measured based on liver stiffness expressed in kilopascals (kPa). For both these measurements, FibroScan^®^ transient elastography (Fibro Scan 501, Echosens, Paris, France) was performed. In the TE test, hepatic steatosis and fibrosis were defined as ≥260 CAP score and ≥5 kPa, respectively (11).

### Transcriptome analysis (mRNA sequencing)

Total RNA was isolated from the liver tissue of the patients. DNA contamination was removed using DNase. Random fragmentation of the DNA strand was performed to sequence the isolated RNA as short reads. After producing the cDNA, a distinct adapter was attached to each end of the cDNA fragment and ligation was carried out. After conducting the polymerase chain reaction (PCR) amplification to obtain the RNA quantity required for sequencing, a 200–400-bp insert was obtained through size selection. Using the reference-based data of aligned reads, transcript assembly was performed using the StringTie program. The level of expression defined by the transcript quantification of each sample was used to generate the expression profile based on the fragments per kilobase of transcript per million mapped reads (FPKM) that represent the normalization values based on the read count, transcript length, and depth of coverage. For cases with known genetic data, Gene Ontology (GO) and Kyoto Encyclopedia of Genes and Genomes (KEGG) databases were used to perform functional annotation and gene-set enrichment analysis for differential gene expression. For single-nucleotide variant (SNV) calling from the RNA-seq data, the mapping of reads to the genomic DNA reference through Spliced Transcripts Alignment to a Reference (STAR) was followed by mark duplication and sorting.

### Blood test

To test the liver function of healthy controls and patients with NAFLD, their blood samples were collected after 8 h of fasting. We used an automated biochemical analyzer (Hitachi Auto Analyzer 747, Japan) to measure the serological parameters related to the body mass index (BMI), alanine aminotransferase (ALT), aspartate aminotransferase (AST), alkaline phosphatase (ALP), cholesterol (CHOL), high-density lipoprotein cholesterol (HDL-C), low-density lipoprotein cholesterol (LDL-C), gamma-glutamyl transferase (GGT), triglycerides (TC), and glucose.

### Cell culture

The HepG2 cell line (Korean Cell Line Bank, KCLB 88065, Korea) was cultured in Dulbecco’s modified Eagle’s medium (DMEM), supplemented with 10% fetal bovine serum (FBS) and 1% penicillin at 37°C in a humidified incubator with 5% CO_2_. The experiments were performed using the same passage cells in cell cultures with 70%–80% confluence.

### mtDNA copy number assay

The HepG2 cells were seeded into a 24-well plate at a density of 2 × 10^5^ cells/well. After 24 h, these cells were treated with 1 nM–1 µM of tamoxifen and left in an incubator for 12 days (37°C, 5% CO_2_). The reagents and media were replaced every 4 days after washing the cells with phosphate-buffered saline (PBS). To extract the total DNA from the cells and peripheral blood using the total DNA extraction kit (BIONICS, Cat. No. DN40200), the DNA concentration was made up to 50 ng/µl after isolating the total DNA. To measure the mtDNA copy number, the TaKaRa Set (no. 7246, Takara Bio USA, Mountain View, CA) was used to perform real-time PCR (Roche LC480). The mtDNA copy number was obtained using the following formula: 2^(nDNAprimerCtValue − mtDNAprimerCtValue)^.

### Mitochondrial ATP inhibition substrate test (ATP assay_Human peripheral blood)

The HepG2 cells were seeded into a 96-well plate at a density of 2 × 10^4^ cells/well. After 24 h, the cells were treated with the media containing 10% serum for 48 h. The cell plate was incubated, and the CellTiter-Glo^®^ 2.0 Assay (Promega, 72052) was performed at room temperature for 30 min. Next, the ATP assay was conducted using the same quantity of the media as that for the CellTiter-Glo^®^ 2.0 Assay, followed by shaking for 2 min. The cells were then incubated at room temperature for 10 min, and their luminescence was measured using Victor 3 (PerkinElmer Victor3, Turku, Finland).

### Mitochondrial ATP inhibition substrate test (ATP assay_HepG2 cell)

The HepG2 cells were seeded into a 96-well plate at a density of 2 × 104 cells/well. After 24 h, the cells were treated with 10 nM–10 µM of doxorubicin. The cell plate and the CellTiter-Glo^®^ 2.0 Assay (Promega, 72052) were incubated at room temperature for 30 min. Then, the ATP assay was treated using the same quantity of the media as that for the CellTiter-Glo^®^ 2.0 Assay, followed by shaking for 2 min. Next, the cells were incubated at room temperature for 10 min, and their luminescence was measured using Victor 3 (PerkinElmer Victor3, Turku, Finland).

### Statistical analysis

All data were expressed as mean ± standard deviation (SD). The data were analyzed using the Student’s *t*-test (for single comparison) or one-way analysis of variance (ANOVA) (for multiple comparisons). Statistical analysis was performed using Prism 7 (GraphPad Software, CA, USA). The level of significance was set to *P* <.05.

## Results

### Mitochondrion-associated transcriptome analysis in NAFLD

**Figure 1:**
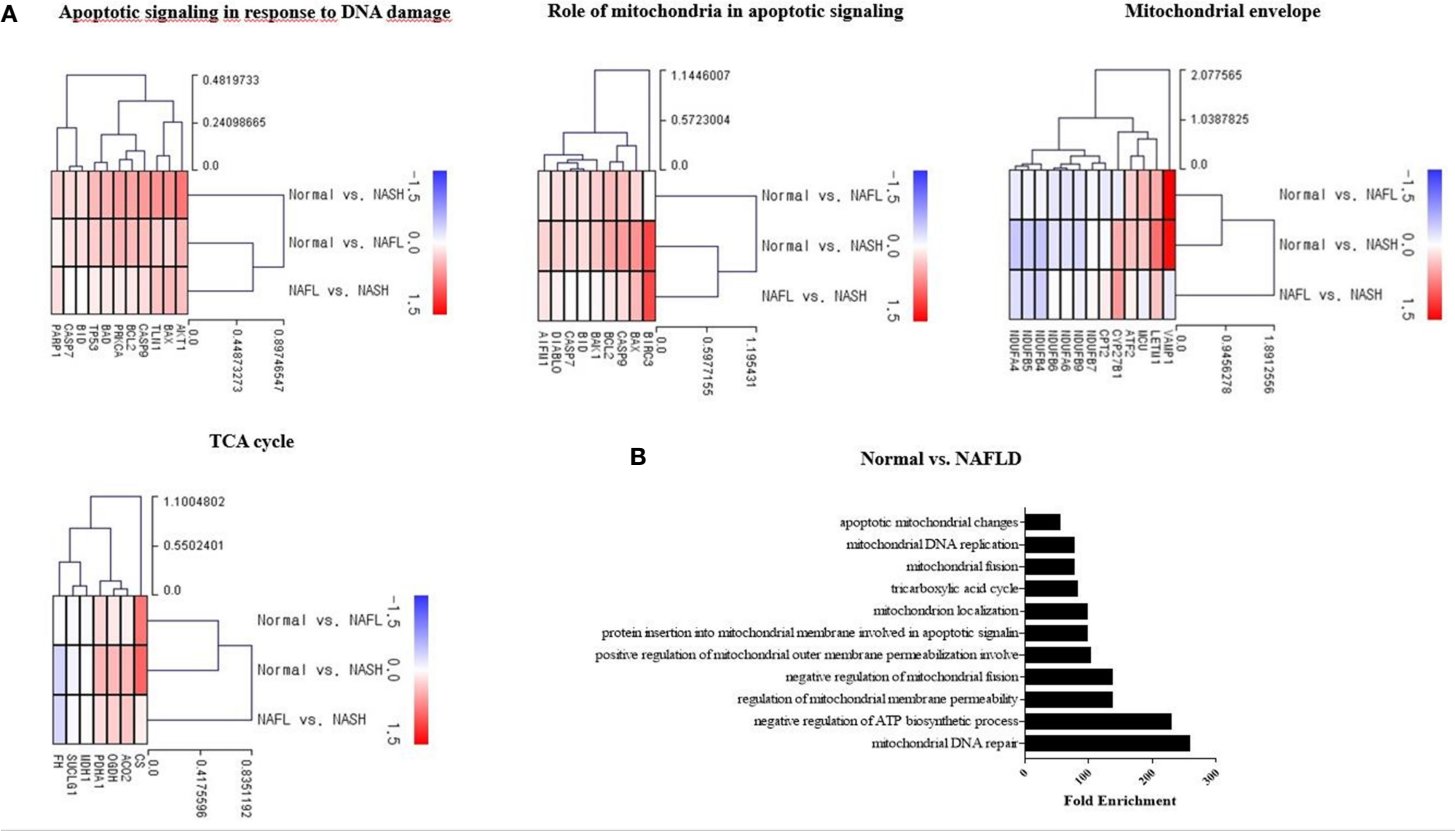
Mitochondria-associated transcriptome analysis in NAFLD. **(A)** Heatmap showing the gene-expression difference in three groups (control, NAFL, and NASH). RNA sequencing was analyzed by the Multiple Experiment Viewer. **(B)** Gene enrichment analysis for DEGs was performed using the Gene Ontology (GO) databases. Functional enrichment analysis based on each module of interest was completed within DAVID, including the GO.

Hepatic mRNA transcriptome analysis was performed for 43 liver-tissue samples obtained from 30 patients with NAFLD (simple steatosis 12, steatohepatitis 18) and 13 healthy controls (**Table 1**). In total, 18,482 genes were identified through transcriptome analysis. The mRNA sequencing data showed that patients with NAFLD had upregulated expression of genes related to pathways such as the tricarboxylic acid (TCA) cycle, apoptotic signaling in response to DNA damage, and role of mitochondria in apoptotic signaling compared with healthy controls. However, some of the mitochondrial envelope-related genes were downregulated compared to the normal ones (**Figure 1A**). The bar chart shows the gene ontology pathway enrichment analysis results (**Figure 1B**).

**Table 1:** Clinical characteristics of the patients (RNA sequencing).

Variable	Normal	NAFL	NASH	P-value	P-value	P-value
		(n= 13)	(n= 12)	(n=18)	(Normal vs. NAFL)	(Normal vs. NASH)	(NAFL vs. NASH)
Age (years)	48.69 ± 8.98	45.33 ± 13.40	44.88 ± 11.94	0.46	0.34	0.92
BMI (kg/m^2^)	23.56 ± 3.50	25.36 ± 3.40	36.49 ± 11.17	0.2	0.0003	0.002
ALT (U/L)	17.38 ± 9.46	32.58 ± 34.09	36.94 ± 30.30	0.13	0.03	0.71
AST (U/L)	19.23 ± 5.13	28.33 ± 23.32	36.11 ± 20.96	0.18	0.008	0.34
ALP (U/L)	76.30 ± 32.76	67.33 ± 21.55	68.22 ± 21.13	0.43	0.41	0.91
CHOL (mmol/L)	161.15 ± 59.04	193.75 ± 37.08	160.77 ± 35.01	0.11	0.98	0.01
HDL-C (mmol/L)	57.45 ± 19.02	62.90 ± 11.84	43.35 ± 11.40	0.4	0.01	<0.0001
LDL-C (mmol/L)	113.30 ± 49.01	121.36 ± 33.99	98.18 ± 25.96	0.66	0.25	0.04
GGT (IU/L)	41.07 ± 71.16	118.91 ± 182.64	58.29 ± 63.19	0.16	0.48	0.2
TG (mmol/L)	105 ± 57.07	121.5 ± 50.92	165.72 ± 83.56	0.45	0.03	0.11
Glucose (mmol/L)	102.84 ± 28.07	93.33 ± 7.48	120.72 ± 40.14	0.26	0.17	0.02

The results are expressed as mean ± SD BMI, body mass index; ALT, alanine aminotransferase; AST, aspartate aminotransferase; ALP, Alkaline Phosphatase; CHOL, cholesterol; HDL-C, high-density lipoprotein cholesterol; LDL-C, low-density lipoprotein cholesterol; GGT, Gamma-Glutamyl Transferase; TG, Triglyceride.

### Mitochondrial dysfunction test

**Figure 2:**
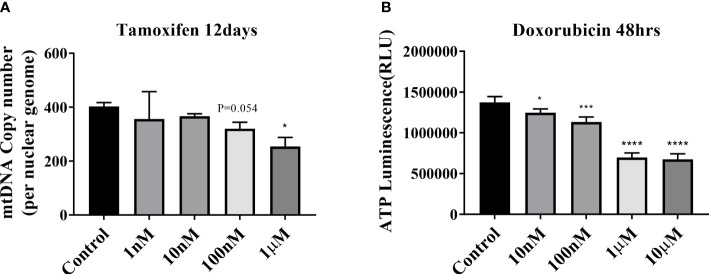
Mitochondrial dysfunction test. **(A)** Results of mitochondrial DNA (mtDNA) copy number treatment of HepG2 cells with tamoxifen for 12 days. *P < .05 compared with the control. **(B)** ATP results obtained by treating HepG2 cells with doxorubicin for 48 h (*P=0.01–0.05; ***P=0.001; ****P < 0.0001).

Tamoxifen and doxorubicin were used as positive controls in the mitochondrial dysfunction test to verify the decrease in the mtDNA copy number and mitochondrial inhibition substrate test (ATP assay test). The mtDNA copy number of HepG2 cells decreased in a dose-dependent manner after treatment with tamoxifen for 8, 12, and 15 days; however, the maximum decrease was observed on 12 days (**Supplementary Figure 1A**; **Figure 2A**). The intracellular ATP concentration also decreased in a dose-dependent manner after the application of doxorubicin for 48 h (**Figure 2B**).

### Baseline characteristics

The mean age of the control group was 56 years, while that of the NAFLD group was 53 years. The BMI of the NAFLD group was significantly higher than that of the control group. The ALP, AST, and ALT levels were also significantly higher in the NAFLD group than those in the control group. The mtDNA copy number and mitochondrial inhibition substrate test (ATP assay test) results were analyzed using the peripheral blood collected from 88 subjects (19 healthy controls and 69 patients with NAFLD) (**Table 2**). The 69 patients were classified into three groups based on their CAP score: CAP score of 260–280: 17 patients; CAP score of 280–300: 13 patients; and CAP score of >300: 39 patients.

**Table 2:** Clinical characteristics of the patients (mtDNA copy number and ATP).

Variable	Normal	NAFLD	P-value
	(n=19)	(n=69)
Age (years)	56.6 ± 13.0	53.8 ± 13.3	0.65561
BMI (kg/m^2^)	22.4 ± 2.6	26.9 ± 3.7	<0.0001
CHOL (mmol/L)	180.7 ± 34.1	182.0 ± 52.2	0.4365
Glucose (mmol/L)	112.2 ± 26.8	130 ± 50.6	0.1607
ALP (U/L)	118.2 ± 89.3	94 ± 34.8	0.0214
AST (U/L)	27.7 ± 8.5	40.9 ± 20.2	0.0023
ALT (U/L)	28.1 ± 16.8	42.7 ± 29.3	0.005
Triglyceride (mmol/L)	143.6 ± 107.0	168.8 ± 111.2	0.2611
HDL-C (mmol/L)	40.6 ± 25.1	17.9 ± 23.7	0.12
GGT (IU/L)	61.5 ± 78.4	54.9 ± 106.6	0.6471
mtDNA copy No.	67.2 ± 21.0	51.4 ± 11.2	<0.0001
ATP assay	2229018 ± 279405.9	1852363.2 ± 205295.2	<0.0001

The results are expressed as mean ± SD BMI, body mass index; ALT, alanine aminotransferase; AST, aspartate aminotransferase; ALP, Alkaline Phosphatase; CHOL, cholesterol; HDL-C, high-density lipoprotein cholesterol; LDL-C, low-density lipoprotein cholesterol; GGT, Gamma-Glutamyl Transferase.

### Mitochondria copy number of peripheral mononuclear cell in NAFLD

**Figure 3:**
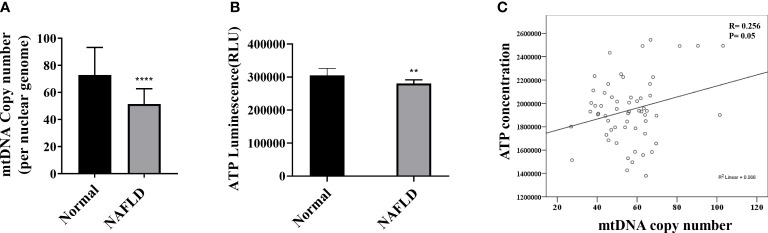
Mitochondrial copy number and mitochondrial ATP inhibition substrate test in NAFLD. **(A)** Results of mitochondrial DNA (mtDNA) copy number decreased in the peripheral blood of NAFLD patients than that in the control. ****P < .0001 compared with the control. **(B)** ATP decreased in the serum isolated from the peripheral blood of NAFLD patients than that in the control (**P=0.001–0.01). **(C)** Positive correlation between the mtDNA copy number and ATP (R = 0.256, P = .05).

**Figure 4:**
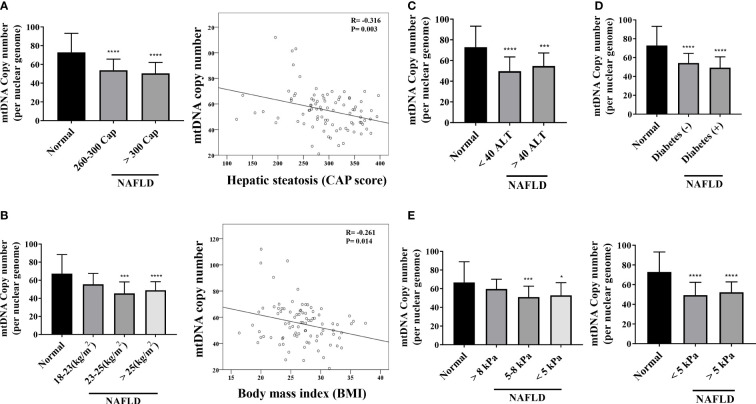
Mitochondrial copy number of peripheral mononuclear cells in NAFLD. **(A)** Results of mitochondrial DNA (mtDNA) copy number based on the hepatic steatosis standard. *P < .0001 compared with the control. There is a negative correlation between the mtDNA copy number and hepatic steatosis (R = -0.316, P = .003). **(B)** Results of mtDNA copy number based on BMI (**P=0.001–0.01; ****P < 0.0001). mtDNA copy number has a negative correlation with BMI (R = -0.261, P = .014). **(C–E)** mtDNA copy number results based on ALT, diabetes, and kPa standards (*P=0.01–0.05; ***P=0.001; ****P < 0.0001).

The mtDNA copy number of peripheral blood mononuclear cells was 1.28-fold lower in patients with NAFLD than that in healthy controls (*P* <.0001) (**Figure 3A**). The mitochondrial ATP inhibition substrate test showed that the intracellular ATP concentration in the serum of NAFLD patients was 1.2-fold less than that in healthy controls (*P* = .0011) (**Figure 3B**). The intracellular ATP concentration showed a positive correlation with the mtDNA copy number (**Figure 3C**). In contrast, the mtDNA copy number in the peripheral blood mononuclear cells showed a negative correlation with the degree of hepatic steatosis and obesity (**Figure 4A, B**). However, diabetes, abnormal ALT levels, and the stage of hepatic fibrosis had no effect on the mtDNA copy number (**Figures 4C–E**).

### Mitochondrial ATP inhibition substrate test (ATP assay)

**Figure 5:**
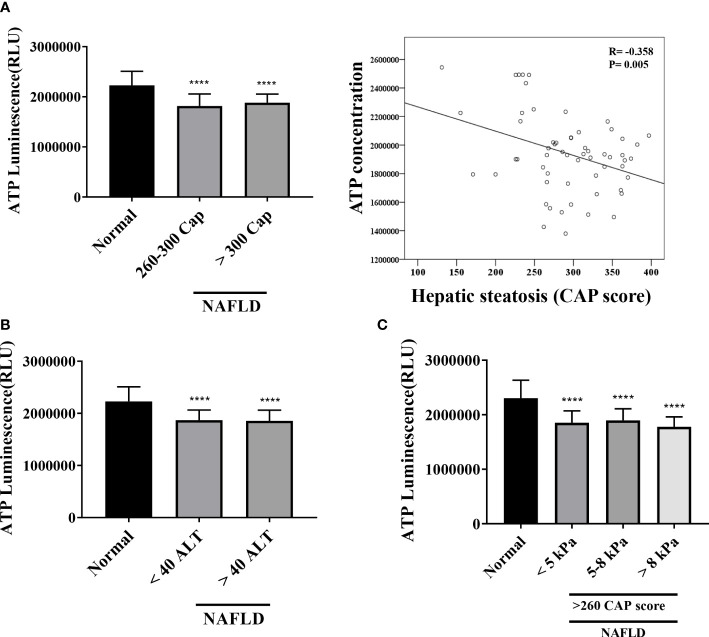
Mitochondrial ATP inhibition substrate test (ATP assay) **(A)** Results of ATP inhibition substrate test according to hepatic steatosis standards. ****P <.0001 compared with the control. ATP inhibition substrate test and hepatic steatosis have a negative correlation (*R* = -0.368, *P* = .005). **(B, C)** ATP inhibition substrate test results based on ALT and kPa standards. ****P <.0001 compared with the control.

The mitochondrial inhibition substrate test results showed a negative correlation with the degree of hepatic steatosis (**Figure 5A**). We confirmed the correlation among the intracellular ATP concentration, ALT, and liver stiffness (in kPa) (**Figures 5B, C**). A statistically significant difference was noted between the control and NAFLD groups. However, no significant difference was observed between the two groups when ALT was ≥40, <40 (P = .8616). Similarly, liver kPa was higher in patients with hepatic fibrosis than that in the normal control group. However, no difference was observed when the three groups were compared based on the kPa values (<5 kPa vs. 5–8 kPa, P = .9188; 5–8 kPa vs. > 8 kPa, P = .4432; <5 kPa vs. >8 kPa; P = .8059).

## Discussion

The hepatic mRNA transcriptome data showed that patients with steatohepatitis had increased expression levels of mitochondrial fusion, apoptotic signal, and mitochondrial envelope genes than those in healthy controls. We determined the mtDNA copy number and performed the mitochondrial ATP inhibition substrate test to determine the biomarkers for evaluating mitochondrial dysfunction in NAFLD. The peripheral blood mononuclear cell mtDNA copy number and mitochondrial ATP inhibition substrate test results were lower in patients with NAFLD than that in the healthy control. Both the mtDNA copy number and ATP concentration showed a negative correlation with the degree of hepatic steatosis.

Transcriptome analysis of the liver tissue of patients with steatohepatitis showed an increase in the mitochondrial envelope and TCA cycle-related gene expression. Patients with NAFL did not show any increase in mitochondrial fusion, apoptotic signal, and mitochondrial envelope-related gene expression, although mitochondria-related gene expression certainly increased in patients with steatohepatitis than that in healthy controls. These data suggest that mitochondrial dysfunction occurs at a relatively late stage of NAFLD, not during the NAFL period.

The HepG2 cells in the serum of patients with NAFLD had a lower concentration of intracellular ATP, implying the presence of a mitochondrial inhibitory substrate in their blood. However, it is not yet clear which component of the blood of these patients induces mitochondrial dysfunction. Considering that the main pathophysiology of NAFLD is apoptosis caused by lipotoxicity, it can be inferred that various constituents of the serum of NAFLD patients may have caused mitochondrial dysfunction. An increase in the amount of free fatty acids in the serum of patients with hepatic steatosis may reduce the mitochondrial function. In addition, an increase in the ceramide contents of the serum of patients with NAFLD may also decrease the mitochondrial function. Ceramide, a sphingolipid, is known to inhibit protein kinase C (PKC). It is involved in apoptosis, inhibition of growth, and aging. A previous study on EDCs investigated the mitochondrial inhibitory function by employing the ATP concentration in cells and suggested the potential influence of mitochondrial inhibitory substrates such as EDCs on the obesity, hyperglycemia, insulin resistance, β-cell function, and inflammatory state (8). However, a correlation between mitochondrial inhibitory substrates and liver dysfunction was found only in female subjects (8).

The peripheral blood mononuclear cell mitochondrial copy number is considered a useful biomarker for mitochondrial dysfunction. The mtDNA copy number is sensitive to oxidative damage, owing to the proximity of mitochondria to areas generating ROS and the lack of histones or DNA-repair systems (12). However, in contrast to the findings of this study, another study reported an increase in the mtDNA copy number in patients with NAFLD compared to that in healthy controls and a decrease in patients with non-alcoholic steatohepatitis (NASH) (13). However, the mtDNA copy number is not reduced in several genetic mitochondrial disorders; instead, it is elevated (14). This observation likely reflects the upregulation of the mtDNA copy number to compensate for poor mitochondrial energetics, including in blood cells (15). This phenomenon is termed compensatory upregulation of the mtDNA copy number (16). This study suggests the critical importance of mitochondrial function in hepatic steatosis. Despite a transient increase in the mtDNA copy number as a compensation mechanism during the early stage of disease, the number decreased again in the case of NASH. The mtDNA copy number is also known to decrease in both diabetic and prediabetic patients. An analysis of the peripheral blood of 125 patients with type II diabetes and 61 healthy controls demonstrated that the mtDNA copy number in the former was lower than that in the latter (17). This difference in the mtDNA copy number remained statistically significant even after adjustments for gender, age, diabetes duration, BMI, and hemoglobin (17). The probability of diabetes increased by 2.8% for each fall in the mtDNA copy number (18). The mtDNA copy number in the peripheral blood of healthy controls and patients with NAFLD showed a correlation between the ATP concentration and BMI and hepatic steatosis. However, the mtDNA copy number and ATP results did not show any correlation with the level of ALT and hepatic fibrosis in patients with NAFLD. This observation is indicative of a problem in measuring the mtDNA copy number in the peripheral blood, owing to the isolation of the total DNA. The peripheral blood is regenerated every 2 weeks, which makes it challenging to assess the level of cumulative oxidative stimuli in chronic conditions. Although RT-PCR can reliably quantify the mtDNA copy number, there are several limitations to its application in clinical practice. The mtDNA copy number is not an absolute measure; it is a relative measure of the number of copies of nuclear DNA in a sample and is subject to variability. Therefore, the mtDNA copy number values cannot be directly compared when running analyses over time in the same individual across studies. In addition, because the number of mitochondria varies with the cell type, the level of the mtDNA copy number depends on the cellular composition of the sample. In the peripheral blood, the mtDNA copy number has a positive correlation with the number of platelets but a negative one with the number of white blood cells (19). Consequently, the measurement of the mtDNA copy number should be normalized to gender, age, platelet count, and white blood cell count.

Several problems need to be resolved before mitochondrial DNA copy number and ATP inhibition testing can be applied in the clinical field. The first is the standardization of the protocol. To standardize the protocol, factors such as reasonable cell lines, positive control, time condition, and precise concentration should be selected. To resolve this problem, we tested more than five cell lines and employed various positive controls at different times to develop an optimal standardization protocol. However, an external validation still needs to be conducted under different laboratory conditions. Second, it is necessary to suggest a cutoff value to define the mitochondrial dysfunction. To meet this guideline, a mitochondrial membrane potential test, electron microscopy, and MitoSOX test were performed to evaluate the mitochondrial function abnormality. Our internal data showed that mitochondrial dysfunction was observed when the mitochondrial ATP inhibition decreased by >30% compared to that in the positive control.

In conclusion, gene expression associated with mitochondrial dysfunction increased in patients with steatohepatitis compared to that in the healthy control group. The mtDNA copy number and mitochondrial ATP were found to have been reduced in the peripheral blood of these patients as compared to those in healthy controls. Hence, mtDNA copy number and mitochondrial ATP inhibition substrate test can be used as biomarkers for determining the mitochondrial dysfunction in patients with NAFLD.

## Data availability statement

The original contributions presented in the study are publicly available. This data can be found here: https://www.ncbi.nlm.nih.gov/sra/?term=PRJNA880989.

## Ethics statement

The studies involving human participants were reviewed and approved by Hanyang University Medical Center. The patients/participants provided their written informed consent to participate in this study.

## Author contributions

DJ and J-HS: designed and supervised the study. JO: conducted the formal analysis. A-HL: assisted in animal studies and the acquisition of the data. HK: analyzed the pathological data. EY: drafted the manuscript. All authors contributed to the article and approved the submitted version.

## Funding

This study was supported by a grant from the Ministry of Food and Drug Safety in 2020 (20183MFDS525). The funding source had no role in study design, implementation, data collection, analysis, and interpretation, or in the preparation, review, or approval of the manuscript.

## Conflict of interest

The authors declare that the research was conducted in the absence of any commercial or financial relationships that could be construed as a potential conflict of interest.

## Publisher’s note

All claims expressed in this article are solely those of the authors and do not necessarily represent those of their affiliated organizations, or those of the publisher, the editors and the reviewers. Any product that may be evaluated in this article, or claim that may be made by its manufacturer, is not guaranteed or endorsed by the publisher.

## References

[1] Younossi ZM Koenig AB Abdelatif D Fazel Y Henry L Wymer M. Global epidemiology of nonalcoholic fatty liver disease – meta–analytic assessment of prevalence, incidence, and outcomes. Hepatology (2016) 64:73–84. doi: 10.1002/hep.28431 26707365

[2] Browning JD Szczepaniak LS Dobbins R Nuremberg P Horton JD Cohen JC. Prevalence of hepatic steatosis in an urban population in the united states: Impact of ethnicity. Hepatology (2004) 40:1387–95. doi: 10.1002/hep.20466 15565570

[3] Osellame LD Blacker TS Duchen MR. Cellular and molecular mechanisms of mitochondrial function. Best Pract Res Clin Endocrinol Metab (2012) 26:711–23. doi: 10.1016/j.beem.2012.05.003 PMC3513836 23168274

[4] Zhou H Du W Li Y Shi C Hu N Ma S. Effects of melatonin on fatty liver disease: the role of NR4A1/DNA–PKcs/p53 pathway, mitochondrial fission, and mitophagy. J Pineal Res (2018) 64 :e12450. doi: 10.1111/jpi.12450 28981157

[5] Wei Y Rector RS Thyfault JP Ibdah JA. Nonalcoholic fatty liver disease and mitochondrial dysfunction. World J Gastroenterol (2008) 14:193–9. doi: 10.3748/wjg.14.193 PMC2675113 18186554

[6] Hodson L McQuaid SE Humphreys SM Milne R Fielding BA Frayn KN. Greater dietary fat oxidation in obese compared with lean men: An adaptive mechanism to prevent liver fat accumulation? Am J Physiol Endocrinol Metab (2010) 299:E584–92. doi: 10.1152/ajpendo.00272.2010 PMC2957864 20628024

[7] Iozzo P Bucci M Roivainen A Nagren K Jarvisalo MJ Kiss J. Fatty acid metabolism in the liver, measured by positron emission tomography, is increased in obese individuals. Gastroenterology (2010) 139:846–56, 56.e1–6. doi: 10.1053/j.gastro.2010.05.039 20685204

[8] Simoes ICM Fontes A Pinton P Zischka H Wieckowski MR. Mitochondria in non–alcoholic fatty liver disease. Int J Biochem Cell Biol (2018) 95:93–9. doi: 10.1016/j.biocel.2017.12.019 29288054

[9] Lee HK Park WH Kang YC Kang S Im S Park S. Serum biomarkers from cell–based assays for AhRL and MIS strongly predicted the future development of diabetes in a large community–based prospective study in Korea. Sci Rep (2020) 10:6339. doi: 10.1038/s41598-020-62550-6 32286339 PMC7156500

[10] Castellani CA Longchamps RJ Sun J Guallar E Arking DE. Thinking outside the nucleus: mitochondrial DNA copy number in health and disease. Mitochondrion (2020) 53:214–23. doi: 10.1016/j.mito.2020.06.004 PMC7375936 32544465

[11] Ajaz S McPhail MJ Gnudi L Trovato FM Mujib S Napoli S. Mitochondrial dysfunction as a mechanistic biomarker in patients with non–alcoholic fatty liver disease (NAFLD). Mitochondrion (2021) 57:119–30. doi: 10.1016/j.mito.2020.12.010 33387664

[12] Chi–Cervera LA Montalvo GI Icaza–Chavez ME Torres–Romero J Arana–Argaez V Ramirez–Camacho M. Clinical relevance of lipid panel and aminotransferases in the context of hepatic steatosis and fibrosis as measured by transient elastography (FibroScan(R)). J Med Biochem (2021) 40:60–6. doi: 10.5937/jomb0-24689 PMC7857856 33584141

[13] Koliaki C Szendroedi J Kaul K Jelenik T Nowotny P Jankowiak F. Adaptation of hepatic mitochondrial function in humans with non–alcoholic fatty liver is lost in steatohepatitis. Cell Metab (2015) 21:739–46. doi: 10.1016/j.cmet.2015.04.004 25955209

[14] Kamfar S Alavian SM Houshmand M Yadegarazari R Seifi Zarei B Khalaj A. Liver mitochondrial DNA copy number and deletion levels may contribute to nonalcoholic fatty liver disease susceptibility. Hepat Mon (2016) 16:e40774. doi: 10.5812/hepatmon.40774 28123441 PMC5237470

[15] Bai RK Wong LJ. Simultaneous detection and quantification of mitochondrial DNA deletion(s), depletion, and over–replication in patients with mitochondrial disease. J Mol Diagn (2005) 7:613–22. doi: 10.1016/S1525-1578(10)60595-8 PMC1867556 16258160

[16] Wei YH Lee CF Lee HC Ma YS Wang CW Lu CY. Increases of mitochondrial mass and mitochondrial genome in association with enhanced oxidative stress in human cells harboring 4,977 BP–deleted mitochondrial DNA. Ann N Y Acad Sci (2001) 928:97–112. doi: 10.1111/j.1749-6632.2001.tb05640.x 11795533

[17] Giordano C Iommarini L Giordano L Maresca A Pisano A Valentino ML. Efficient mitochondrial biogenesis drives incomplete penetrance in leber’s hereditary optic neuropathy. Brain (2014) 137:335–53. doi: 10.1093/brain/awt343 PMC3914475 24369379

[18] Picard M. Blood mitochondrial DNA copy number: What are we counting? Mitochondrion (2021) 60:1–11. doi: 10.1016/j.mito.2021.06.010 34157430 PMC8464495

[19] Latini A Borgiani P De Benedittis G D’Amato C Greco C Lauro D. Mitochondrial DNA copy number in peripheral blood is reduced in type 2 diabetes patients with polyneuropathy and associated with a MIR499A gene polymorphism. DNA Cell Biol (2020) 39:1467–72. doi: 10.1089/dna.2019.5326 32311290

