# Supplementary material for: Peripheral blood mononuclear cell mitochondrial copy number and adenosine triphosphate inhibition test in NAFLD
Source: Front Endocrinol (Lausanne). 2022 Oct 24;13:967848. doi: 10.3389/fendo.2022.967848 (PMC9637714; doi:10.3389/fendo.2022.967848)
Supplement: Supplementary file 1 [file DataSheet_1.docx]

**
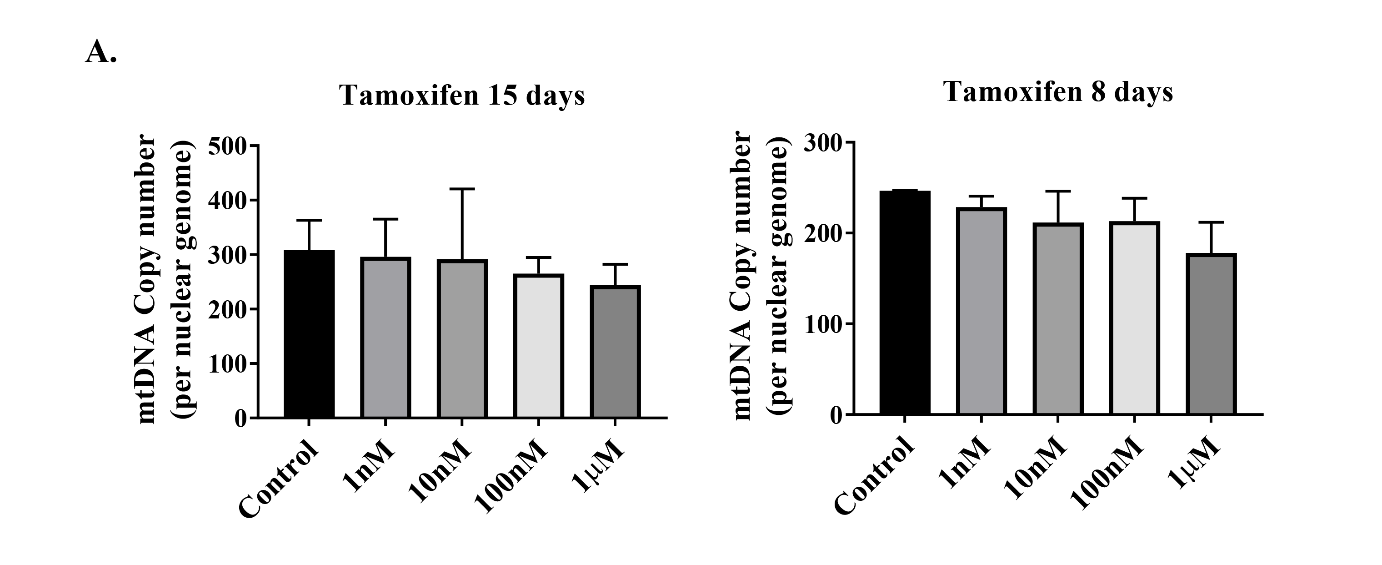
Supplement figure 1: Establishment of mitochondrial dysfunction test** A. This is the result of treating the date of drug treatment differently to establish the mtDNA copy number measurement method.
